# Rare Monogenic Diseases: Molecular Pathophysiology and Novel Therapies

**DOI:** 10.3390/ijms23126525

**Published:** 2022-06-10

**Authors:** Ivano Condò

**Affiliations:** Department of Biomedicine and Prevention, University of Rome Tor Vergata, 00133 Rome, Italy; ivano.condo@uniroma2.it

A rare disease is defined by its low prevalence in the general population. Although the fixed threshold slightly varies between different countries, a specific disorder is usually considered to be rare when it affects less than 50–60 in 100,000 individuals. The inherited defects originating from single gene mutations characterize the vast panorama of rare monogenic diseases. More than 4000 monogenic mutations account for at least 80% of all rare diseases, involving a broad spectrum of clinical features and pathophysiological mechanisms. Strikingly, rare monogenic diseases often appear during childhood and remain largely untreatable, underscoring the hard challenges in biology and medicine to identify the underlying molecular mechanisms and to develop effective therapies.

The Special Issue “Rare Monogenic Diseases: Molecular Pathophysiology and Novel Therapies” provides a collection of original research articles and systematic reviews focused on diverse conditions stemming from a pathogenic single gene mutation. However, we are aware of at least 6000 monogenic phenotypes and clearly this cannot be an exhaustive collection of all the rare monogenic diseases. The included articles cover current progress in pathogenesis mechanisms and experimental therapeutics concerning such diverse conditions as neurodegenerative or neurodevelopmental disorders, lysosomal storage diseases, coagulation disorders, hemoglobinopathies, kidney diseases and cancer.

Among rare monogenic diseases, close to 70% of these disorders affects the nervous system. In particular, predominant phenotypic manifestations are represented by neurodevelopmental and neurodegenerative alterations. Loi et al. present preclinical data supporting a new therapeutical strategy for the disease caused by mutations in X-linked cyclin-dependent kinase-like 5 (*CDKL5*) gene [1]. CDKL5 deficiency disorder (CDD) is characterized by defects in neuronal development and survival, a condition with no available therapy. Because single inhibitors able to target GSK-3β kinase and histone deacetylases (HDAC) have been shown to have neuroprotective effects in CDD mice, the authors demonstrate in vitro and in vivo the efficacy of a novel combinatorial therapy using Compound 11 (C11), a synthetic GSK-3β/HDAC dual inhibitor [1]. Trinucleotide repeat disorders, a group of neuropathological conditions caused by abnormal expansion of natural triplet repeats in a specific gene, are the focus of two systematic reviews. The expansion of CAG trinucleotide repeats within the coding sequence of huntingtin gene (*HTT*) generates the elongation of polyglutamine sequence in the mutated HTT protein and triggers Huntington’s disease (HD), a neurodegenerative disorder examined by Tomczyk et al. [2]. This review describes the complex network of molecular and cellular changes affecting purine metabolism and signaling in HD. Based on the summarized dysfunctions, the authors analyze and propose potential therapeutic strategies able to target intracellular and extracellular adenosine levels [2]. On the other hand, the pathogenic expansion of CGG triplets occurs in the 5’ non-coding sequence of the Fragile X Mental Retardation 1 (*FMR1*) gene and leads to various early or late-onset Fragile-X-associated syndromes. Valor et al. review the molecular pathogenic mechanisms involved in the adult syndromes, a spectrum of gain-of-function disorders [3]. The authors analyze in detail the mechanistic roles of mutant *FMR1* mRNA (riboCGG), *FMR1*-derived long non-coding RNAs (lncRNAs), RNA-binding proteins and *FMR1*-derived cryptic polyglycine peptide (FMRpolyG). Moreover, an interesting description of established and potential biomarkers for adult Fragile-X-associated syndromes is provided [3].

Lysosomal storage disorders (LSDs) are metabolic diseases that result from genetic enzyme deficiencies. Although more than 50 genes are involved in specific pathogenesis, most storage disorders are systemic because of the ubiquitous functions of lysosomes. Currently, approved drugs for some LSDs include recombinant proteins for enzyme replacement therapy (ERT) and small molecules acting as pharmacological chaperone therapy (PCT). Unfortunately, these approaches present important therapeutic limitations. Total or partial loss of lysosomal α-galactosidase A (GLA) enzyme activity results in Fabry disease (FD), a multisystemic X-linked disorder. Naturally, aggressive disease manifestations develop more frequently in male FD patients. However, depending on the random X chromosome inactivation, female patients may exhibit milder signs and symptoms. Modrego et al. provide a systematic overview of the knowledge stemming from the countless mutations affecting the *GLA* gene in FD patients [4]. By analyzing the structure-function relationships of GLA enzyme, these authors illustrate the potential use of recombinant GLA mutants characterized by increased enzyme activity, improved protein stability or lower immunogenicity. Altogether, the proposed approach may generate novel therapeutics to promptly improve ERT and PCT for the treatment of FD [4]. Mucopolysaccharidosis type II (MPS II) is another X-linked LSD caused by a mutation in the gene encoding iduronate 2-sulphatase (*IDS*). This multisystemic disorder is partially treatable with ERT. However, the recombinant enzyme cannot cross the blood–brain barrier, thus no improvement is possible in central nervous system of MPS II patients with severe forms. Zapolnik and Pyrkosz evaluate the progresses of gene therapy as future alternative to cure MPS II patients [5]. By describing successes and failures of MPS II preclinical and clinical studies based on retroviruses, lentiviruses, adeno-associated viruses and genome editing technology, this review looks at possible achievements of ongoing and upcoming MPS II clinical trials centered on gene therapies [5].

Monogenic blood disorders may be associated with abnormalities in a protein either soluble in blood plasma or present within blood cells. For instance, mutations in the genes encoding coagulation factors critically affect the blood clotting mechanism and lead to bleeding disorders. The review by Rodríguez-Merchán et al. illustrates the promises of gene therapy in the context of hemophilia A, arising from mutations in coagulation factor VIII gene, and hemophilia B, triggered by mutations in coagulation factor IX gene [6]. This paper also inspects the main clinical trials on hemophilia patients to discuss opportunities and limits of current adenoviral vector-mediated gene transfer protocols [6]. Parahemophilia or Owren’s disease, is an ultra-rare coagulation disorder associated with mutations in the factor V gene. Bernal et al. describe the molecular study performed to characterize two patients’ families and the consequent discovery of a new mutation leading to severe factor Va deficiency [7]. β-thalassemia is instead a blood cells disorder, linked to mutations of the β-globin gene and reduced expression of the encoded adult hemoglobin (HbA) protein in erythrocytes. The original article by Zuccato et al. focuses on the induction of fetal hemoglobin (HbF) as experimental therapy to overcome HbA deficiency in β-thalassemia [8]. This study demonstrates that Cinchonidine and Quinidine, two natural alkaloids from Cinchona plants, are able to induce HbF expression in affected cells derived from β-thalassemia patients and to potentiate the similar effects of sirolimus, a drug currently under clinical evaluation to treat this hemoglobinopathy [8].

The selective expression or the particular role of specific genes in a single tissue explains the appearance of organ-specific inherited diseases. This is the case of genetic disorders of the kidney, which include dominant and recessive forms of cystic diseases, and renal tubulopathies. Mutations in polycystin-1 (*PKD1*) or -2 (*PKD2*) genes lead to autosomal-dominant polycystic kidney disease (ADPKD), whose gender-dependent phenotype was analyzed in the study by Talbi et al. [9]. These results, obtained in mice lacking PKD1 expression, show the involvement of intracellular Ca2+ levels in the more severe phenotype affecting male ADPKD animals. Altogether, identification of the molecular mechanisms underlying enhanced Ca2+ signaling and proliferation in cells from male kidneys may contribute to develop novel therapeutics for ADPKD [9]. The autosomal-recessive form of polycystic kidney disease (ARPKD) mostly arises from defects in the gene named polycystic kidney and hepatic disease 1 (*PKHD1*), whereas a minority of cases is linked to a second causative gene *DZIP1L*. To examine the still unclear molecular pathophysiology of ARPKD, Cordido et al. recapitulate known molecular disease mechanisms and possible therapeutic approaches, from cellular and animal models to clinical trials [10]. The knowledge of ARPKD pathogenic pathways, involving the epidermal growth factor receptor (EGFR) axis, the production of adenylyl cyclase adenosine 3′,5′-cyclic monophosphate (cAMP) and the activation of several protein kinases, begins to stimulate possible pharmacological interventions [10]. Inherited loss of function in various electrolyte transport proteins located along the nephron leads to two types of kidney tubulopathy with overlapping clinical symptoms: Gitelman and Bartter syndromes. The review by Nuñez-Gonzalez et al. aims to explain the different molecular basis of these difficult to diagnose monogenic syndromes. Moreover, the authors provide an overview of current therapeutic approaches and highlight the presence of common and specific options for Gitelman and Bartter patients [11].

Finally, the disparate group of rare monogenic diseases also include well-known genetic mutations that directly cause inherited cancers. These kinds of neoplasia occur by two classic types of germline mutations: inactivating alterations in a tumor suppressor gene or activating variants in a proto-oncogene. Multiple endocrine neoplasia type 1 (MEN1) is triggered by mutations in the tumor suppressor gene *MEN1* encoding for menin, a nuclear protein involved in broad transcriptional regulation. Given the absence of a direct genotype-phenotype correlation in tumorigenesis affecting patients with identical *MEN1* mutations, the review by Marini and Brandi explores the involvement of epigenetic regulations [12]. In light of the molecular effects of microRNA-24 (miR-24) on menin expression, the authors discuss a possible role of this miRNA in MEN1 development and progression. The potential use of RNA therapeutics able to target miR-24 in MEN1 patients is examined as well [12].

Overall, this collection of articles underlines how molecular therapies for a monogenic disorder may target either directly the mutated gene-product or the associated pathway(s). Indeed, as understanding of molecular mechanisms improves, novel therapeutics targeting different disease pathways become conceivable. By providing insights into specific molecular defects and broadly applicable therapies, these contributions may be a valuable resource to stimulate future scientific advances in the field of genetic diseases.
